# Supplementary material for: Mapping the evolution of fertility support policies in China: A content and instrumental analysis
Source: PLoS One. 2025 Oct 9;20(10):e0332137. doi: 10.1371/journal.pone.0332137 (PMC12510515; doi:10.1371/journal.pone.0332137)
Supplement: S1 Appendix — (ZIP) [file pone.0332137.s001.zip › S1 Appendix. 226 original policy documents/5-国家人口计生委关于印发《全国“十一五”人口和计划生育事业发展规划》的通知(FBM-CLI-4-83633).doc]

国家人口计生委关于印发《全国“十一五”人口和计划生育事业发展规划》的通知

**发布部门：** [国家人口和计划生育委员会(含国家计划生育委员会)(已撤销)](https://www.pkulaw.com/law/chl?Aggs.IssueDepartment=60304) [机构沿革](javascript:void(0))

**发布日期：**2006.12.18

**实施日期：**2006.12.18

**时效性：** [现行有效](https://www.pkulaw.com/law/chl?Aggs.TimelinessDic=01)

**效力级别：** [部门工作文件](https://www.pkulaw.com/law/chl?Aggs.EffectivenessDic=XE0303)

**法规类别：** [人口计划管理](https://www.pkulaw.com/law/chl?Aggs.Category=09701) [计划生育管理](https://www.pkulaw.com/law/chl?Aggs.Category=09705)

国家人口计生委关于印发《全国“十一五”人口和计划生育事业发展规划》的通知

各省、自治区、直辖市人口计生委，计划单列市、新疆生产建设兵团人口计生委，解放军、武警部队人口和计划生育领导小组办公室，中直机关、中央国家机关人口计生委，委机关各单位，各直属、挂靠单位：（国人口发〔2006〕141号）
　　《全国“十一五”人口和计划生育事业发展规划》已经国家人口和计划生育委员会党组会议和主任会议审议通过。现印发各地区、各单位贯彻执行。
　　本规划是国家级专项规划，是指导“十一五”时期我国人口和计划生育事业发展的纲领性文件。各级人口计生部门和各单位要认真学习贯彻《中共中央国务院[关于全面加强人口和计划生育工作统筹解决人口问题的决定](https://www.pkulaw.com/chl/5e7b01010a4102ffbdfb.html)》精神，全面贯彻本规划确立的指导思想和原则，紧扣事业发展目标，抓好主要任务和重点工作的落实，组织好重大工程项目的实施。要根据本规划，制定和完善本地区人口和计划生育事业发展规划、规划实施方案及年度计划并组织实施。严格落实规划确定的约束性指标，不得突破。为促进规划的顺利实施，国家人口计生委将加强监督检查和监测评估，并将落实本规划情况作为衡量各地区、各单位工作的重要依据。
　　“十一五”时期，人口和计划生育事业进入稳定低生育水平、统筹解决人口问题、促进人的全面发展的新阶段。人口和计划生育系统要以高度的历史责任感和使命感，全面贯彻落实科学发展观和构建社会主义和谐社会重大战略思想，积极探索中国特色统筹解决人口问题的道路，进一步开创人口和计划生育工作的新局面。

　　二○○六年十二月十八日

　　附：

全国“十一五”人口和计划生育事业发展规划
（国家人口和计划生育委员会）

目 录

　　第一章　“十五”时期成就与“十一五”时期面临的形势
　　第一节　主要成就与经验
　　第二节　形势与挑战
　　第二章　指导思想、原则与目标
　　第一节　指导思想
　　第二节　基本原则
　　第三节　发展目标
　　第三章　主要任务
　　第一节　稳定低生育水平
　　第二节　大力提高出生人口素质和群众生殖健康水平
　　第三节　综合治理出生人口性别比偏高问题
　　第四节　加强流动人口管理与服务
　　第五节　积极应对人口老龄化
　　第四章　重点工作
　　第一节　深化综合改革，建立健全工作新机制
　　第二节　全面推行依法行政，实现部门工作法治化
　　第三节　完善利益导向机制，增进计划生育家庭福利
　　第四节　加强宣传教育，建设先进人口文化和新型生育文化
　　第五节　加大科研开发力度，增强自主创新能力
　　第六节　加强技术服务体系建设，提高服务能力和水平
　　第七节　建立流动人口管理服务新体制，维护流动人口合法权益
　　第八节　实施新农村新家庭计划，提高农村人口生活质量
　　第九节　充分利用社会资源，创新社区管理服务模式
　　第十节　深化人口发展战略研究，增强科学决策能力
　　第十一节　深化统计改革，推进人口信息化建设
　　第十二节　扩大对外开放，增进人口与发展领域的国际交流与合作
　　第五章　保障措施
　　第一节　坚持和完善党政第一把手亲自抓、负总责制度
　　第二节　健全人口和计划生育宏观调控体系和综合治理机制
　　第三节　建立人口和计划生育财政投入保障机制
　　第四节　建设高素质职业化的人口和计划生育队伍
　　第五节　建立健全规划实施机制
　　本规划根据《[中华人民共和国国民经济和社会发展第十一个五年规划纲要](https://www.pkulaw.com/chl/62f50194a99f4ce8bdfb.html)》、《人口发展“十一五”和2020年规划》和《中共中央国务院[关于全面加强人口和计划生育工作统筹解决人口问题的决定](https://www.pkulaw.com/chl/5e7b01010a4102ffbdfb.html)》编制，主要阐明“十一五”时期人口和计划生育事业发展目标和工作重点，指导和规范各级政府行为，调动各方面积极性和创造性，全面落实科学发展观，推进国家人口发展战略，确保人口和计划生育事业持续健康发展，促进经济社会和人的全面发展。

　第一章　“十五”时期成就与“十一五”时期面临的形势

　第一节　主要成就与经验

“十五”时期是我国人口和计划生育事业承前启后、与时俱进的五年。面对复杂的人口和计划生育形势，在党中央、国务院的正确领导下，各级党委政府、人口和计划生育部门求真务实，开拓创新，顺利实现预定的发展目标。低生育水平继续稳定，年增长率保持在7‰以下，2005年末全国总人口（不含香港、澳门特别行政区和台湾省，下同）为130756万人；人口和计划生育事业法制化、信息化步伐加快；机构实现更名、部门职能得到拓展，人口发展战略研究取得重大成果；综合改革不断深化，利益导向机制建设取得突破性进展；计划生育宣传教育和优质服务工作深入开展，群众满意度明显提高；人事制度改革稳步推进，干部队伍整体素质明显提高；人口与发展领域的国际交流与合作进一步加强。这些都为“十一五”时期人口和计划生育事业发展奠定了良好基础。面对未来，人口和计划生育事业已经进入了“稳定低生育水平，统筹解决人口问题，促进人的全面发展”的新阶段。

| 专栏1“十五”时期主要进展情况 | | | | | | |
| --- | --- | --- | --- | --- | --- | --- |
| 指　标 | 2000年 | 2001年 | 2002年 | 2003年 | 2004年 | 2005年 |
| 总人口(万人) | 126743 | 127627 | 128453 | 129227 | 129988 | 130756 |
| 自然增长率(‰) | 7.58 | 6.95 | 6.45 | 6.01 | 5.87 | 5.89 |
| 出生人口（万人） | 1771 | 1702 | 1647 | 1599 | 1593 | 1617 |
| 自然增长（万人） | 957 | 884 | 826 | 774 | 761 | 768 |

　　“十五”时期人口和计划生育事业发展的基本经验是，全面落实科学发展观，坚持用改革的思路和发展的方法解决前进中遇到的新情况、新问题；坚持人口与发展综合决策，充分发挥党委和政府的主导作用；坚持长期实行计划生育基本国策，稳定和完善人口政策和生育政策；坚持党政第一把手亲自抓、负总责，动员社会力量综合治理人口问题；坚持以人的全面发展为中心，不断创新工作思路、机制和方法；坚持国家指导与群众自愿、宣传教育与利益导向、行政管理与群众工作相结合，重视发挥非政府组织的作用；坚持因地制宜、分类指导、整体推进，推动事业全面协调可持续发展；坚持人口与发展领域的对外开放，树立负责任人口大国的良好形象。

　第二节　形势与挑战

“十一五”时期，人口和计划生育事业迎来新的发展机遇，也面临着前所未有的严峻挑战。党中央提出的全面落实科学发展观、构建社会主义和谐社会以及建设社会主义新农村等重大战略部署，为全面推进人口和计划生育事业指明了方向；综合国力不断增强，社会发展投入逐步加大，为增进人民群众福利、进一步提高计划生育生殖健康水平提供了坚实的物质基础；市场化、工业化、城镇化、信息化及国际化进程加快，将对人们的生产、生活和生育方式产生重大影响，进一步促进群众生育观念的转变；政府职能转变加快，法制体系不断完备，人民群众法制观念不断增强，将进一步改善人口和计划生育依法行政的社会环境；农村公共卫生体系和计划生育服务网络的建设，将为人口和计划生育事业发展提供日益雄厚的技术支撑；在长期实践中形成的行政部门、服务机构、自治组织、群众团体相结合的管理服务网络和“特别能吃苦、特别能战斗、特别能奉献、善于做群众工作”的工作队伍，为确保新时期人口和计划生育事业持续快速健康发展提供了强有力的人才支持和组织保障。
　　同时，应清醒地看到，在经济转轨、社会转型的关键时期，我国人口和计划生育事业面临的人口形势更加复杂，承担的任务更加艰巨。低生育水平仍不稳定，人口结构性问题日益凸现，出生人口素质亟待提高，流动人口管理服务需要加强。人口总量基数大且持续增长。未来十几年，20-29岁生育旺盛期妇女数量将形成一个高峰，9000多万独生子女陆续进入生育年龄，全国人口将以年均800-1000万人的速度增长。群众生育意愿与国家政策要求还存在较大差距，工作稍有放松，生育水平就会反弹。出生人口性别比偏高问题尚未得到有效解决。出生缺陷发生率仍然较高，群众生殖健康状况有待进一步改善。流动迁移人口规模庞大并将持续相当长的时期。区域间、城乡间人口发展差异明显。人口老龄化速度加快，将对经济社会发展产生复杂而深远的影响。
　　制约人口和计划生育事业发展的深层次矛盾和问题依然存在。稳定低生育水平的激励约束机制、提高出生人口素质的综合干预机制、出生人口性别比偏高问题的综合治理机制、计划生育家庭的社会保障机制、公民计划生育生殖健康权利的保障机制及流动人口的管理服务机制等制度建设仍然滞后，统筹解决人口问题的综合决策机制、宏观调控体系和公共投入机制仍不健全。一些地方党政领导对人口和计划生育事业的重视程度有所减弱，人口和计划生育部门的工作思路、机制和方法以及队伍的管理服务能力与新形势新任务的要求还不相适应。一些地方基层基础工作比较薄弱，少数地方出现了工作滑坡和生育水平反弹的迹象。
　　面对机遇和挑战，必须以高度的历史责任感和使命感，认真贯彻落实《中共中央国务院[关于全面加强人口和计划生育工作统筹解决人口问题的决定](https://www.pkulaw.com/chl/5e7b01010a4102ffbdfb.html)》，知难而进，开拓创新，积极探索中国特色统筹解决人口问题的道路，牢牢把握战略主动权，进一步开创人口和计划生育工作新局面。

　第二章　指导思想、原则与目标

　第一节　指导思想

以邓小平理论和“三个代表”重要思想为指导，全面落实科学发展观，按照构建社会主义和谐社会和建设社会主义新农村的要求，坚持以人为本，树立优先投资于人的全面发展的战略理念，以稳定和完善人口政策和生育政策为前提，以深化综合改革、健全工作新机制为动力，以增强公共服务和社会管理能力为基础，以推进人口与发展科学决策和综合治理为保障，把人口和计划生育事业切实转入稳定低生育水平、统筹解决人口问题、促进人的全面发展的轨道。

　第二节　基本原则

--坚持以人为本。从解决关系人民群众切身利益的现实问题出发，切实保障群众计划生育生殖健康基本权益，不断提高出生人口素质和计划生育家庭的社会保障与福利水平，促进人的全面发展。
　　--坚持人口与发展科学决策。坚定不移地推行计划生育基本国策和改革开放，统筹人口自身以及人口与经济社会资源环境的协调发展和可持续发展，把人口和计划生育事业纳入国民经济和社会发展的总体规划，进行统筹协调，整体推进。
　　--坚持人口问题的综合治理。综合运用经济、社会、法律、行政等手段，形成党政领导、部门配合、社会参与、齐抓共管的综合治理格局。
　　--坚持以改革促发展、以创新求实效。加快管理体制和工作机制改革步伐，推进工作思路、机制和方法的创新，注重创新能力建设，实现工作思路由单纯控制数量为主向在稳定低生育水平的基础上统筹解决人口问题转变，工作方式由行政制约为主向依法管理、优质服务、综合施治转变。
　　--坚持因地制宜、分类指导。城市、东部发达地区率先在统筹解决人口问题方面取得重要进展，东部欠发达农村、东北农村和中部农村地区注重建立稳定低生育水平的长效机制，西部农村地区继续控制人口增长，实现人口和计划生育事业城乡、区域协调发展。

　第三节　发展目标

普遍建立人口和计划生育工作新机制，全面提升公共服务和社会管理能力，初步形成统筹解决人口问题的良好局面，确保低生育水平继续稳定，出生人口素质明显提高，出生人口性别比升高势头得到有效遏制，人民群众普遍享有基本的计划生育、生殖健康和家庭保健服务，计划生育家庭生产、生活状况明显改善，权益得到有效保障。

| 专栏2“十一五”时期主要发展目标与指标 | | | |
| --- | --- | --- | --- |
| 指　标 | 2005年 | 2010年 | 属　性 |
| 总人口（亿人） | 13.0756 | 13.6000 | 约束性指标 |
| 总和生育率 | 1.8左右 | | 约束性指标 |
| 自然增长率(‰) | 6-9‰ | | 约束性指标 |
| 出生人口素质 | 明显提高 | | 预期性指标 |
| 出生人口性别比 | 升高势头得到有效遏制 | | 预期性指标 |
| 流动育龄人口在流入地服务覆盖率 | 85% | | 预期性指标 |
| 人均事业经费（元） | 18.27 | 30 | 约束性指标 |

　第三章　主要任务

　第一节　稳定低生育水平

坚持稳定和完善现行生育政策不动摇。认真执行《[中华人民共和国人口与计划生育法](https://www.pkulaw.com/chl/252f7ce51132230bbdfb.html)》及相关法律法规，保持现行生育政策的稳定性和连续性。进一步完善有利于贯彻落实现行生育政策的经济、社会、法律、行政等措施。坚持党政一把手亲自抓、负总责不动摇。全面落实人口和计划生育工作目标管理责任制，严格执行“一票否决”。坚持稳定人口和计划生育工作机构、队伍不动摇。在县、乡机构改革中，确保负责计划生育工作的机构和人员。明确人口和计划生育服务机构为财政全额拨款的社会公益性事业单位，不得随意撤并和改变。确保基层“有人管事、有钱办事”。确保避孕节育措施的落实。坚持不断创新人口和计划生育工作体制、机制、手段和方法不动摇。建立和完善稳定低生育水平的利益导向机制。稳定低生育水平的重点在农村，难点是农村人口和流动人口。切实加强农村计划生育管理服务能力建设，深入开展计划生育村民自治。

　第二节　大力提高出生人口素质和群众生殖健康水平

全面实施出生缺陷一级干预。科学制定提高出生人口素质的规划及行动计划，加强出生缺陷干预和生殖保健服务能力建设。研究制定我国常见出生缺陷的病因及发病机理，开发临床筛查、检测、预防新技术和孕前风险评估方法。以社区和家庭为基础开展出生缺陷干预工作，逐步建立具有人口和计划生育系统特色的出生缺陷干预模式。普及优生优育和生殖健康知识，积极开展孕前医学检查，预防和控制先天性感染、遗传性和环境因素对出生人口健康的影响。开展人口早期教育和独生子女社会性发展培养工作，为人的终身发展创造良好开端。立足社区和家庭，面向健康、亚健康人群，积极探索各具特色的生殖健康促进模式，满足人民群众日益增长的计划生育、生殖健康和家庭保健需求。

　第三节　综合治理出生人口性别比偏高问题

广泛深入开展关爱女孩行动，综合治理出生人口性别比偏高问题。建立党政负责、部门配合、群众参与的标本兼治的工作机制。认真贯彻计划生育、男女平等基本国策，以消除性别歧视为重点，深入开展全民宣传倡导，增强社会性别平等意识，引导人们树立“生男生女顺其自然”、“生男生女一样好”等科学、文明、进步的婚育观念。贯彻同等优先的原则，制定和实施有利于女孩健康成长和妇女发展的社会经济政策。对农村计划生育女儿户给予奖励，在扶贫济困、慈善救助、贴息贷款、就业安排、项目扶持中对计划生育女儿户予以倾斜。鼓励男到女家落户，依法保护妇女的宅基地、房屋等继承权和土地承包权等权益。严禁非医学需要的胎儿性别鉴定和选择性别的人工终止妊娠。建立B超检查和人工终止妊娠登记、孕情检测、孕产过程管理等制度。完善执业资格认证和B超使用准入制度。对终止妊娠药品和促排卵药品实行严格的处方管理。运用法律手段，严厉打击非法实施胎儿性别鉴定和选择性别人工终止妊娠的行为，依法严惩溺、弃、残害女婴和拐卖、绑架妇女儿童的犯罪活动和歧视、虐待生育女婴的妇女等违法行为，保护妇女儿童合法权益。探索建立跨地区、跨部门的出生性别监控系统和选择性别终止妊娠综合监管制度。实施举报制度，加强社会监督。把出生人口性别比偏高问题综合治理纳入目标管理责任制，科学考核。

　第四节　加强流动人口管理与服务

加快建立和完善以流入地管理为主、流出地和流入地协调配合的流动人口计划生育管理服务体系。将流动人口纳入流入地管理服务，维护流动人口实行计划生育的合法权益。加强部门间流动人口计划生育工作综合协调，推进跨区域流动人口计划生育管理服务的协作。加强流动人口统计和信息化建设，为引导人口有序流动和合理分布提供及时、准确的信息服务。贯彻国家区域发展、主体功能区和促进城镇化的战略部署，与相关部门协作，加强人口流动与分布的政策研究，探索建立引导人口有序流动和合理分布的长效机制。

　第五节　积极应对人口老龄化

开展人口发展的前瞻性研究，科学把握人口老龄化趋势及其影响因素，与相关部门密切合作，研究制定应对措施和政策。在全社会倡导和推动“积极老龄社会”行动，弘扬敬老风尚，营造老有所养、老有所乐、老有所为的社会氛围。全面实施农村计划生育家庭奖励扶助制度，积极探索建立计划生育独生子女和双女户家庭养老保障制度。鼓励有条件的地方为计划生育独生子女、双女户家庭的老年人提供预防保健、权益保障、精神慰籍和生活照料等方面的服务。

　第四章　重点工作

　第一节　深化综合改革，建立健全工作新机制

全面深化综合改革，着重推动理论创新、制度创新、科技创新、能力创新和载体创新，在全国大多数地区基本建立起“依法管理、村（居）民自治、优质服务、政策推动、综合治理”的工作新机制。国家和省级人口计生委的工作职能要切实转到“研究战略、制定规则、创造环境、依法行政”上来。构建特色鲜明的行政部门、服务机构、自治组织、群众团体互联、互动、互补的公共管理服务体系。充分发挥工会、共青团、妇联、计生协、人口福利基金会等非政府组织的积极作用。

　第二节　全面推行依法行政，实现部门工作法治化

认真贯彻落实《[全面推进依法行政实施纲要](https://www.pkulaw.com/chl/61052ca84cd0d03fbdfb.html)》。制定实施《人口和计划生育系统依法行政“十一五”规划》，探索建立科学化、民主化和规范化的行政决策机制。进一步完善人口和计划生育法律法规体系，为统筹解决人口问题提供法律保障。制定、落实“五五”普法规划，加强《[人口与计划生育法](https://www.pkulaw.com/chl/252f7ce51132230bbdfb.html)》、《[流动人口计划生育工作管理办法](https://www.pkulaw.com/chl/c8062869d0fb3652bdfb.html)》、《[计划生育技术服务管理条例](https://www.pkulaw.com/chl/b5b4877bc6cd96cabdfb.html)》和《[社会抚养费征收管理办法](https://www.pkulaw.com/chl/f03446d289d2b59ebdfb.html)》等法律法规的宣传贯彻，增强干部群众的法律意识和法制观念，提高干部依法行政能力和水平。全面推行行政执法责任制，坚持正确执法、文明执法，维护和保障群众实行计划生育的合法权益。完善层级监督机制，提高行政监督效能，逐步拓宽基层民主管理、民主监督渠道。

　第三节　完善利益导向机制，增进计划生育家庭福利

落实法定的各项计划生育奖励优先优惠政策，不断创新利益导向制度。全面实施农村计划生育家庭奖励扶助制度和“少生快富”工程。落实独生子女父母奖励制度和计划生育基本服务项目免费制度，探索建立独生子女伤残死亡家庭困难扶助、农村计划生育长效节育措施奖励、农村计划生育纯女家庭奖励、农村计划生育节育手术保险、城市计划生育夫妇退休一次性奖励等制度。协调有关部门在制定相关经济社会政策时，对计划生育家庭特别是农村独生子女家庭和双女户家庭给予适当优惠。巩固和扩大“幸福工程-救助贫困母亲行动”、“春蕾计划”的成果。广泛动员社会各界力量，大力支持以计划生育协会为主导开展的“生育关怀行动”。探索建立政府资助、企业捐赠、社会募集、非政府组织运作的人口和计划生育公益基金。

　第四节　加强宣传教育，建设先进人口文化和新型生育文化

坚持宣传教育为主的指导方针，构建大宣传、大联合的宣传教育新格局。围绕统筹解决人口问题，强化人口和计划生育理论宣传、群众宣传、新闻宣传和社会宣传，广泛、深入、持久地开展婚育新风进万家活动和关爱女孩行动，引导、帮助和促进群众婚育观念的转变。通过报刊、广播、电视和互联网等多种媒体，开展群众喜闻乐见的宣传教育活动，传播计划生育、优生优育、生殖健康和预防性病、艾滋病等科学知识，增强群众自我保健意识和能力，培育文明健康的生活方式，促进家庭幸福和社会和谐。加强各级人口和计划生育宣传教育工作机构、网络和队伍建设，建立健全宣传教育工作体系和工作机制。加强各级党政干部人口理论教育和各级党校、行政学院等的人口理论教学与科研工作。加强与教育部门配合，在小学高年级和中学以上学校普遍开设人口和计划生育、青春期性与生殖健康的课程。进一步加大对农村特别是中西部地区和贫困地区农村宣传教育工作的支持力度。在城市社区建设和新农村建设中，利用各种文化场所和宣传途径，开展群众广泛参与的先进人口文化和新型生育文化建设活动。
　　探索建立以素质教育为核心，以人的全面发展为目标的早期教育和独生子女培养模式。在全国建立一批“中国人口早期教育暨独生子女培养示范区”，运用科学理论与方法，对胎儿和0-3岁婴幼儿实施科学的胎教和早期教育，对其家长和看护人员进行科学的育儿指导、咨询和服务，为全国婴幼儿早期保育、教育和独生子女的社会性培养提供示范。

　第五节　加大科研开发力度，增强自主创新能力

构建以需求为导向、产学研相结合的计划生育生殖健康科技创新体系。加快科研院所体制改革，建立现代院所制度。发挥国家级人口和计划生育科研机构在科技创新中的龙头作用，发挥省级人口和计划生育科研机构在应用研究、新技术新产品推广、指导基层技术服务等方面的骨干作用。加强科技基地建设，新建一批部委级重点实验室和工程技术中心。引导企业成为科技创新的主体。充分发挥传统中医药优势，运用生物技术、信息技术和新材料技术的最新成果，开展计划生育生殖健康领域重点课题的联合攻关。加大影响出生缺陷的生物遗传、社会环境、不良生活方式等重大危险因素研究和评估力度，加强计划生育生殖健康基础研究和应用研究，围绕生育、节育、不育，研发一批避孕节育、优生优育和生殖保健关键技术和产品。积极推广使用计划生育生殖健康新技术、新产品。制定和实施生殖健康产业发展规划，引导企业加大对新技术、新产品研发投入，优化产业发展环境，创新产业发展机制，促进生殖健康产业发展。

　第六节　加强技术服务体系建设，提高服务能力和水平

全面实施《“十一五”期间农村基层计划生育服务体系建设规划》。切实加强县、乡计划生育服务站标准化和规范化建设，加强县级服务机构改造、扩建，在中西部地区建设一批计划生育中心乡（镇）服务机构。在中西部地区和东部困难地区县（市、区）普遍配备和有计划地更新计划生育流动服务车，并配备开展计划生育生殖健康和优生优育技术服务所必须的手术和检查筛选设备。形成以县级服务站为龙头、乡（镇）中心服务站为骨干、普通乡（镇）服务站为依托、村服务室为基础、流动服务车为纽带，人口计生部门和卫生部门密切合作、功能互补、资源共享的农村人口和计划生育技术服务体系。制定省和地（市）级避孕药具管理站建设的国家标准，建立便捷、高效的避孕药具配送系统。
　　全面实施计划生育生殖健康促进计划。加强面向群众的宣传、咨询和指导，普及优生优育知识。开展出生缺陷流行病学调查和出生缺陷孕前风险评估，鼓励婚前、孕前医学检查。制定出生缺陷干预实施指南和服务规范，做好优生检查、孕期服务。建立和完善病残儿医学鉴定报告制度。开展避孕指导、孕前咨询、随访转诊，有条件的社区为病残儿家庭提供优生咨询和指导，并做好孕期服务。深入开展优质服务先进县（市、区）创建活动。继续推进避孕方法知情选择。加强避孕药具不良反应监测评估。积极实施妇科病普查防治工作，逐步建立包括流动人口在内的已婚育龄妇女生殖健康档案。加强生殖道感染干预。面向育龄群众开展旨在增强生殖保健意识和自我保健能力的科普知识教育。开展全民预防性病艾滋病知识普及教育、咨询服务和安全套推广活动。建立人口和计划生育技术服务质量保证体系，加强规范化管理，规范技术服务机构市场准入和资格认证。

　第七节　建立流动人口管理服务新体制，维护流动人口合法权益

将流动人口纳入流入地计划生育经常性管理和服务范围，享有与户籍人口同等待遇。完善流动人口计划生育管理服务网络，配备相应的工作人员。按照常住人口规模配备专（兼）职管理与服务人员。进城务工人员计划生育管理和服务经费纳入流入地各级财政预算。实行以流入地为主的目标管理责任考核。把流动人口计划生育管理和服务纳入社区流动人口综合管理体系。强化社区流动人口登记制度，实现流动人口信息适时变动、异地查询和跟踪管理。建立流动人口计划生育的区域协作制度和部门合作制度。大力宣传流动人口计划生育生殖健康的各项法律法规和政策，定期开展法律法规和政策落实情况的督查，大力开展流动人口计划生育的各项维权活动。

　第八节　实施新农村新家庭计划，提高农村人口生活质量

充分发挥人口和计划生育管理服务体系在建设社会主义新农村中的作用，实施新农村新家庭计划。以人的全面发展为中心，以家庭需求为导向，以提高家庭的生育质量、生命质量和生活质量为主线，促进农村家庭特别是计划生育家庭的生育文明、权利保障、能力发展和福利改善。大力开展小康生活、人口健康、人口文化、社会保障和民主建设方面的促进工作。建立和完善综合信息管理平台，加强社会管理和公共服务信息的采集、传播与反馈。加强农村人口思想文化阵地建设，倡导科学、文明、进步的社会新风尚。开展农村人口健康促进活动，促进家庭幸福。动员、组织群众参加多方面的技能培训，提高农民群众就业能力。开展相关政策法规的咨询培训服务，增强农民群众维护合法权益的意识。

　第九节　充分利用社会资源，创新社区管理服务模式

以社区群众需求为导向，加强部门合作，充分利用和有效整合行政管理资源、社会资源和基础设施资源，增强社区人口和计划生育管理服务能力。加强乡（镇）、街道服务站、村（居）服务室在宣传教育、政策咨询、行政事务办理、信息采集、技术服务、避孕药具发放等方面的职能建设。创新服务模式，推行网格化管理，切实加强城市住宅小区和流动人口的计划生育管理和服务。推进社区信息网络建设与应用，立足常住人口进行信息采集、维护更新和统计考评。充分发挥计划生育协会等群众组织和志愿者在社区人口和计划生育管理服务中的作用。

　第十节　深化人口发展战略研究，增强科学决策能力

坚持政府牵头、部门协作、专家为主、社会参与，建立健全人口发展战略研究长效机制。有条件的地方可建立人口发展战略研究专项基金，探索和完善行之有效的战略研究模式。围绕与科学发展观、社会主义和谐社会建设相适应的人口发展指标体系，社会主义新农村建设与人口和计划生育工作，应对人口老龄化，引导人口有序流动和合理分布，重大区域人口发展战略，生态屏障、功能区划与人口发展，人口政策，人口发展模式的国际比较等重大问题，深入开展课题研究。加强对区域和省级人口发展战略研究的协调指导，鼓励有条件的地方建立区域性人口发展研究基地或中心，形成国家、区域及省级人口发展战略研究合理分工、密切合作、功能整合、整体推进的良好局面。

　第十一节　深化统计改革，推进人口信息化建设

完善统计指标体系。探索建立以人的全面发展为中心，全面反映人口数量、素质、结构、分布及其与经济、社会、资源、环境协调发展和可持续发展的综合统计指标体系。改进统计手段和方式。完善专项调查、工作记录、定期报表有机结合的统计调查体系。落实数据公告制度，定期公布人口和计划生育数据和信息。
　　加强对人口数据的定点监测和评估工作，建立人口发展综合监测评估预警系统。推进人口信息的综合开发利用和有效整合。建立部门间人口信息共享机制，逐步实现互联互通和业务协同。加快国家和省级人口宏观管理与决策信息系统建设，在国家和部分省建立人口数据的快速采集、动态监测和系统分析体系，初步形成计划生育、就业、流动人口管理服务、人口老龄化等人口发展领域的辅助决策支持体系框架，为科学研究和决策提供信息支撑。以实用、好用为标准，建设和完善重点业务应用系统。按照全国统一的数据库结构和代码标准，兼顾各地的业务需求，整合各地的育龄妇女信息系统（WIS），加强奖励扶助和“少生快富”工程信息系统、全国流动人口计划生育信息交换平台和人事统计等事业统计信息系统的建设。整合部门信息资源，实现流动人口信息共享。进一步完善人口计生委办公信息系统，建立和完善面向公众的信息服务系统，积极推进网上政务公开和在线公共服务。完善信息工作制度，建立信息化标准体系和安全保障体系。持续深入开展信息岗位练兵活动，培养、锻炼一支掌握现代信息技术、熟悉业务应用系统的专业工作队伍。

　第十二节　扩大对外开放，增进人口与发展领域的国际交流与合作

贯彻国际人口与发展大会行动纲领，推进实现联合国千年发展目标。继续加强同人口与发展南南合作伙伴组织成员国政府间合作，加强与发展中国家的政策对话、经验交流和人员培训。将人口和计划生育及生殖健康领域的南南合作纳入国家南南合作总体框架。增进避孕药具和生殖健康产品方面的南南合作，努力把避孕药具和生殖健康产品推向国际市场。加强同发达国家政府间的对话与合作。加强对外宣传，建立突发事件宣传应急机制。继续巩固和发展与国际组织的合作，拓展与联合国人口基金等相关机构的合作渠道。推动与国外相关科研机构的学术交流与合作，引进国外先进的管理、技术和经验。加强项目管理，探索建立国际项目为国内工作服务的运作机制。充分利用中国计划生育协会、中国人口福利基金会等非政府组织的独特优势，拓宽国际交流渠道和合作领域。

专栏3　“十一五”时期人口和计划生育主要工作目标1、在全国85%以上的地区基本建立工 作新机制。 2、实现人口和计划生育部门工作法治化。 3、全国基本实现优质服务，30%以上的县（市、区）达到国家优质服务标准，40%以上的县 （市、区）达到省级优质服务标准。 4、单项的国家利益导向政策对象资格确认误差率控制在5‰以内。 5、全国县乡服务站基本达到国家建设标准。 6、新型婚育观念和新型生育文化基本形成。 7、综合避孕率达85%，育龄群众生殖保健基础知识普及率达80%，育龄妇女生殖道感染干预 覆盖面达80%。 8、以人群和社区为基础的出生缺陷一级干预覆盖面达60%以上。 9、县乡建立育龄妇女信息系统，90%以上已婚育龄妇女纳入信息管理，初步实现流动人口信 息的异地交换与共享。

专栏4　　“十一五”时期重大工程项目-“甘露计划”1、计划生育管理服务能力建设工程： 主要包括计划生育服务体系和干部职业化发展与培训项目、流动人口计划生育管理示范区 项目。 2、计划生育家庭奖扶工程：主要包括农村部分计划生育家庭奖励扶助制度、西部地区计划 生育“少生快富”工程、独生子女死亡伤残家庭困难扶助制度、农村计划生育手术平安保险 、农村计划生育长效措施奖励制度等。 3、出生人口性别比综合治理工程：主要包括婚育新风进万家活动、关爱女孩行动、农村计 划生育纯女户父母奖励制度、出生人口性别综合监控系统等。 4、计划生育生殖健康促进工程：主要包括生殖健康科学知识的宣传普及、避孕措施知情选 择、免费计划生育服务项目、孕情和避孕措施的跟踪服务和生殖道感染干预、预防艾滋病和 安全套推广、出生缺陷一级干预、避孕药具不良反应监测等项目。 5、新农村新家庭建设项目：主要包括生产发展服务、文化教育促进、人口健康促进、基层 民主建设、家庭社会保障等方面内容。 6、人口宏观管理与决策信息系统：建立和完善相关的技术标准和管理规范；依托国家电子 政务网络和公网，实现人口计生部门与试点省、信息直报点、其他相关政府部门之间的互联 互通；建立育龄妇女信息、人口和计划生育决策支持等数据库，建设人口信息采集等应用系 统；建设和完善相关的安全保障系统和运行维护系统。 7、人口和计划生育国际合作项目：主要包括南南国家人力资源开发和产品技术交流援助、 中国/联合国人口基金第六周期的生殖健康计划生育项目等。

　第五章　保障措施

　第一节　坚持和完善党政第一把手亲自抓、负总责制度

建立科学的考核评估体系，落实目标管理责任制。把各级党政领导落实人口和计划生育工作情况作为衡量政绩和选拔奖惩的重要内容，对工作失职的要追究责任，确保认识到位、责任到位、措施到位、投入到位。探索建立保障责任落实、客观评价工作水平、有利于调动基层积极性的考核评估机制，促进考核工作规范化、科学化和制度化。改革目标管理责任制考核办法，对党政领导、相关部门和计划生育部门分别进行考核，实行“一票否决”制度。本规划确定的约束性指标，要纳入各地区的综合评价和绩效考核，分解落实到有关部门。积极倡导公众参与，发挥公众评价和社会监督的作用。

　第二节　健全人口和计划生育宏观调控体系和综合治理机制

建立部际协调机制，统筹协调人口发展战略、规划、政策及与人口发展相关经济社会政策的制定和实施。将人口和计划生育工作纳入科学发展综合考评体系和社会主义新农村建设总体规划，协调相关部门在制定经济社会政策时向计划生育家庭适当倾斜。明确各部门承担人口和计划生育的职责，建立区域间人口发展合作与交流制度，形成部门联手、区域合作、齐抓共管、综合治理的统筹解决人口问题的局面。

　第三节　建立人口和计划生育财政投入保障机制

加大财政投入力度，确保计划生育经常性工作、免费技术服务基本项目、法定的各项奖励优惠政策、县乡服务网络和队伍建设所需经费落实到位。财政对人口和计划生育事业投入增长幅度要高于经常性财政收入增长幅度。各级财政要逐年增加投入，到2010年，全国人均人口和计划生育事业费在“十五”期末人均10元的基础上增加到22元，继续安排“十五”期间已将社会抚养费、乡镇统筹费纳入财政预算的人口和计划生育事业费支出人均8元，届时，各级财政投入人口和计划生育事业费达到人均30元。加大对经济欠发达、条件艰苦、工作困难地区的财政支持力度。加强财务管理和监督，建立科学、规范的财务管理制度，提高资金使用效率，严肃查处财政专项资金和社会抚养费征收、管理、使用中出现的问题。

　第四节　建设高素质职业化的人口和计划生育队伍

深化人事制度改革，建立健全人口和计划生育工作激励约束机制。建立科学的考核评估体系，对任务完成情况实行绩效考核。完善人员准入、选拔任用、引进培养、交流退出机制。加强县、乡人口和计划生育执法监督，切实履行流动人口计划生育管理服务、出生人口性别比综合治理、社会抚养费征收等法定职责。进一步加强队伍职业道德和党风廉政建设，积极构建教育、制度、监督并重的惩治和预防腐败体系，深入开展民主评议政风行风活动。总结先进经验与典型，表彰先进集体和先进个人。继续开展争做“新时期最可爱的人”的活动。发挥“中华人口奖”的激励作用，鼓励人口发展领域的综合管理和研究创新。
　　建设一支高素质、职业化的管理服务队伍。探索建立教育培训长效机制，形成以职业教育为基础、任职培训、岗位培训和继续教育为主体的终身教育网络。重点加强县级以上人口和计划生育部门负责人的业务培训和县、乡计划生育技术服务人员的技术培训。优化技术服务人才结构，不断提高医学、心理学、公共卫生学、社会工作学等专业人才比例。对队伍进行职业分类，制定职业标准和相应的考核评估指标。加强职业教育教材、师资队伍和基地网络建设。建立相关职业资格认证制度和任职资格评定体系。按规定落实基层工作人员的工资待遇和养老保险。

　第五节　建立健全规划实施机制

建立衔接统一的层级规划体系。本规划确定的主要目标和指标纳入各地国民经济和社会发展规划与年度计划。各级人口和计划生育部门要根据《人口发展“十一五”和2020年规划》和本规划，制定本地区人口和计划生育事业发展规划、规划实施方案及年度计划并组织实施。本规划确定的约束性指标按年度分解到各省、自治区、直辖市，严格落实，不得突破。
　　建立规划实施的监测、评估机制和民主决策、公众参与机制。加强对规划执行情况的监测、评估、督导和工程项目动态跟踪及实施效果评估。建立规划中期和末期评估制度，根据变化情况，对规划适时修订和完善。充分发挥人口专家委员会和科技专家委员会的作用，加强规划和工程项目的前期研究、咨询、论证和评估工作。采取多种方式和渠道，鼓励公众参与，增强规划实施及修编过程的公开性和透明度。

© 北大法宝：（[www.pkulaw.com](https://www.pkulaw.com/)) 专业提供法律信息、法学知识和法律软件领域各类解决方案。北大法宝为您提供丰富的参考资料，正式引用法规条文时请与标准文本核对。
欢迎查看所有[产品和服务](https://www.pkulaw.com/helps/2.html)。法宝快讯：[如何快速找到您需要的检索结果？](https://www.pkulaw.com/helps/70.html)[法宝](https://www.pkulaw.com/helps/69.html)V6有何新特色？

扫描二维码阅读原文

**原文链接：**<https://www.pkulaw.com/chl/5f57e09cfb871b72bdfb.html>
